# Immunological findings of West Caucasian bat virus in an accidental host

**DOI:** 10.1128/jvi.01914-24

**Published:** 2025-01-23

**Authors:** Martina Castellan, Gianpiero Zamperin, Greta Foiani, Maira Zorzan, Maria Francesca Priore, Petra Drzewnioková, Erica Melchiotti, Marta Vascellari, Isabella Monne, Sergio Crovella, Stefania Leopardi, Paola De Benedictis

**Affiliations:** 1 Laboratory for Emerging Viral Zoonoses, WOAH Reference Laboratory for Rabies, FAO and National Reference Centre for Rabies, Department for Research and Innovation, Istituto Zooprofilattico Sperimentale delle Venezie 83372 , Legnaro, Italy; 2 Viral Genomics and Transcriptomics Laboratory, Department for Research and Innovation, Istituto Zooprofilattico Sperimentale delle Venezie 83372 , Legnaro, Italy; 3 Laboratory of Histopathology, Istituto Zooprofilattico Sperimentale delle Venezie 83372 , Legnaro, Italy; 4 Laboratory of Animal Research Center (LARC), Qatar University 61780 , Doha, Qatar; St. Jude Children's Research Hospital, Memphis, Tennessee, USA

**Keywords:** transcriptomic analysis, rabies virus (RABV), Duvenhage virus (DUVV), West Caucasian bat virus (WCBV), Syrian hamster, host response

## Abstract

The *Lyssavirus* genus includes seventeen viral species able to cause rabies, an acute and almost invariably fatal encephalomyelitis of mammals. Rabies virus (RABV), which represents the type species of the genus, is a multi-host pathogen that over the years has undergone multiple events of host-switching, thus occupying several geographical and ecological niches. In contrast, non-RABV lyssaviruses are mainly confined within a single natural host with rare spillover events. In this scenario, unveiling the mechanisms underlying the host immune response against a virus is crucial to understand the dynamics of infection and to predict the probability of colonization/adaptation to a new target species. Presently, the host response to lyssaviruses has only been partially explored, with the majority of data extrapolated from RABV infection. West Caucasian bat virus (WCBV), a divergent lyssavirus, has recently been associated with a spillover event to a domestic cat, raising concern about the risks to public health due to the circulation of the virus in its natural host. Through this study we have investigated the immune response determined by the WCBV versus two widely known lyssaviruses. We selected the Syrian hamster as representative of an accidental host, and chose the intramuscular route in order to mimic the natural infection. In hamsters, WCBV was highly pathogenic, determining 100% lethality and mild encephalitis. In comparison with Duvenhage virus (DUVV) and RABV, we found that WCBV displayed an intermediate ability to promote cellular antiviral response, produce pro-inflammatory cytokines, and recruit and activate lymphocytes in the hamsters’ central nervous system.

**IMPORTANCE:**

Although all lyssaviruses cause fatal encephalomyelitis in mammals, they display a different host tropism and pathogenicity, with the ecology of Rabies virus (RABV) continually evolving and adapting to new host species. In 2020, West Caucasian bat virus (WCBV) was identified as the causative agent of rabies in a domestic cat in Italy. This event raised concerns about its public health consequences, due to the absence of biologicals against the infection. Our study investigates the host immune response triggered by WCBV in comparison with a pathogenic strain of RABV and the low pathogenic Duvenhage lyssavirus (DUVV), as a proxy to understand the mechanisms leading to lyssavirus spillover and pathogenicity. We overall confirm that previous evidence indicating an inverse relationship between lyssavirus pathogenicity and immune response is applicable for WCBV as well. Importantly, this work represents the first transcriptomic analysis of the WCBV interaction in the central nervous system with an accidental host.

## INTRODUCTION

Viruses belonging to the *Lyssavirus* genus (family Rhabdoviridae, subfamily Alpharhabdovirinae) are neurotropic pathogens and the causative agents of rabies, an acute encephalomyelitis of mammals (1, 2). Among the 17 lyssaviruses currently recognized by the International Committee on the Taxonomy of Viruses (3), Rabies virus (RABV) is the representative member of the species and is mainly transmitted through the exposure of non-intact skin or mucosa to the saliva of an infected animal (4, 5). Upon transmission, RABV replicates locally in the wound before spreading from the neuromuscular junction to motor neurons, reaching the peripheral nervous system (PNS) (5–7). Viral particles enter the primary motor neurons by budding from muscle cells into the synaptic cleft of neuromuscular junctions and travel through the PNS toward the central nervous system (CNS) via microtubule-dependent retrograde axonal transport (8–12). RABV infection of the CNS is characterized by the inhibition of the host immune response; as such, after the onset of clinical symptoms, major inflammatory lesions are barely identifiable in a RABV-infected brain (13–15).

RABV is a multi-host pathogen that has established multiple transmission cycles in different host species, thus occupying several geographical and ecological niches (16, 17), with frequent spillover events to humans (18, 19). Indeed, dog-mediated RABV infection still causes most human and animal rabies cases worldwide (20) with an estimated 59,000 deaths per year, mainly in Asia and Africa (21–24). Non-RABV lyssaviruses have restricted geographical and host ranges, being mostly associated to bat species, with rare spillover events in the absence of further transmission in the accidental hosts (25–28). Due to the few characterized cases in nature and difficulties in investigating lyssaviruses natural reservoirs, little is known about the pathogenicity and mechanisms behind the elicitation of the immune response by the non-RABV lyssaviruses, with most information directly translated from RABV knowledge (29–32). In this context, the interaction between a pathogen and its host and the ways the host possibly reacts to the infection determine the development and severity of symptoms, as well as its final outcome. Thus, understanding the molecular connections between a virus and the host immune system represents a key determinant not only to understand and predict the disease dynamics within the host, but also to define the risk for animal and public health associated with the likelihood of spillover and further transmission within an accidental host species. Until recently, host response against lyssaviruses has only partially been unveiled for RABV, assuming all members of the lyssavirus genus perform the same. However, increasing evidence highlights the weakness of such an assumption, and indicates that lyssaviruses likely differ in their pathogenicity and stimulation of the host immune response (33–37). It is also worth mentioning that RABV strains can act very differently from each other and have higher or lower levels of pathogenicity in the occasional host (19, 38, 39), just like other members of phylogroup I showed a reduced ability to develop rabid symptoms in the experimental models. As such, Duvenhage virus (DUVV) has shown little or non-neuroinvasiveness in mice in different experimental settings (40, 41) and is able to elicit a strong antiviral response and activation of the interferon (IFN) signaling pathway in mice, similarly to a cell-attenuated RABV strain (42). Nevertheless, DUVV has been associated with spillover events, with three human deaths reported so far (26, 43, 44). Similarly, West Caucasian bat virus (WCBV) was first detected in 2002 in a bent-winged bat (*Miniopterus schreibersii*) in Russia (45) and no further identification had been observed until recently, neither in bats nor in non-flying mammals, posing doubts about its actual circulation in nature and capability of such a divergent virus to infect accidental host species. In 2020, WCBV was confirmed able to cause symptomatic rabies in non-flying mammals after spilling on a cat in Italy (46).

In this study, we have investigated the pathogen-host interaction and immune responses determined by the 2020 WCBV (46) in an accidental host, using the Syrian hamsters as an animal model, and compared them to those observed following RABV and DUVV infections.

## MATERIALS AND METHODS

### Viruses

The Italian WCBV strain (EvaG Ref-SKU: 025V-04866), detected in a symptomatic cat in 2020 in Central Italy (46), and the RABV (Cosmopolitan lineage; EvaG Ref-SKU: 025V-04868) identified in 2019 from a human case acquired abroad (47) were both amplified in newborn albino mice (backup stock), bred *in house* at the IZSVe. DUVV belonged to the historical repository of the Istituto Zooprofilattico Sperimentale delle Venezie (IZSVe), originally isolated from a Miniopterus bat in South Africa and subsequently amplified in newborn mice, as described for the other two lyssaviruses (backup stock). For the purpose of the present study, the backup stocks were further replicated once in mice and the collected brains were homogenated, five times diluted in phosphate-buffered saline (PBS), using a Tissue Lyzer (Qiagen). The homogenate was further clarified from tissue debris with a centrifugation step and the supernatants from all the infected homogenized brains were collected to obtain the viral working stock. Viral sequencing was undertaken using 1 million reads in the Illumina platform and a metagenomics approach, as detailed elsewhere (48). Full genome sequences of DUVV, WCBV, and RABV are available under GenBank accession numbers PP869293.1, MZ501949.1, and OQ787037.1, respectively. All the working viral batches were sequenced as previously described (49) to assess the accuracy of the sequences. See Table S1 for genetic comparisons among reference strains and working batches of WCBV and DUVV.

### Animal experiment

The study involved 25 eight-week-old female Syrian hamsters divided into five experimental groups as follows: two groups of *n* = 5 animals each, intramuscularly injected with either RABV or WCBV, respectively; one group of *n* = 9 animals intramuscularly injected with DUVV; and two groups of *n* = 3 control animals each (Mock). The first infection of five animals with DUVV led to three rabid hamsters only, forcing us to repeat the *in vivo* challenge with the other four animals (only two of them developed rabies symptoms). DUVV was unable to determine a 100% lethal infection, with four out of nine animals surviving the challenge and not displaying any neurological signs. The results obtained with the DUVV-infected group of nine individuals were coherent with the information available from the literature that indicate poor pathogenicity of the virus in mice (40, 41). Animals were acclimatized 7 days prior to infection in individual cages (BCU-2 Rat Sealed Negative Pressure IVC, Allentown Inc) in biosafety level 3 (BSL3) animal facilities, following national and international regulations on the welfare of laboratory animals. Procedures were all performed under general anesthesia that was guaranteed by isoflurane-based gas anesthesia (up to 230 pL/dm^3^), induced by the use of an induction chamber. Animals were inoculated intramuscularly (gastrocnemius muscles, 100 µL each limb) with 1:5 clarified mice brain homogenate in PBS containing 5.86 Log MICLD_50_/mL (or 7,18^5^ MICLD/mL) of each virus. Mock animals were similarly inoculated with a sterile PBS solution. We followed and registered the animals’ clinical conditions for up to 60 days after the infection and sacrificed them at the onset of severe symptoms. More specifically, animals were monitored at least twice a day for the development of rabies clinical signs, with daily recording of their weight and the evaluation of their physical features, activity, posture, and neurological symptoms. Signs included depression, loss of weight between 10% and 20%, mono or bilateral paralysis of the hind limb and dyspnea were considered severe and sufficient as humanitarian threshold and animals were euthanized through CO_2_ inhalation under general anesthesia. Mock animals were euthanized concomitantly to the infected ones. Whole brains were collected immediately after death and divided into two parts following the sagittal plane. Hemibrains were either fixed in 10% neutral-buffered formalin for 48 h for histological examination or placed in RNA later (Thermo Fisher) for 24–48 h at +4°C and then stored at −80°C after medium removal.

### Histology, immunohistochemistry and immunofluorescence

Formalin-fixed hemibrains were routinely processed for histology and embedded in paraffin. 4 µm-thick parasagittal brain sections were stained with hematoxylin and eosin (H&E) for histological examination. Inflammatory changes were scored based on an adapted scoring system described elsewhere (50, 51). Perivascular cuffs were counted in four consecutive 20× fields, starting from more inflamed areas, in three mainly affected macro-regions (pons/medulla oblongata, midbrain/thalamus, and cerebellum). In addition, each perivascular cuff was scored on a scale of 1–3 (1 = mild; 2 = moderate; 3 = severe; Table S2). The final histopathological score was defined as the weighted sum of the number of perivascular cuffs multiplied by the severity score of each cuff. Slides were analyzed, and images were taken using a Leica DM4 B light microscope with a DFC450 C Microscope Digital Camera and the software Leica Application Suite V4.13 (Leica Microsystems, Wetzlar, Germany). A 4 µm serial parasagittal brain sections of formalin-fixed, paraffin-embedded tissue were submitted to automated immunohistochemistry (IHC) (Discovery ULTRA system, Roche, Ventana Medical Systems Inc., Tucson, AZ, USA) with the primary antibodies anti-CD3 (Dako, Agilent Technologies, Glostrup, Denmark), PAX5 (Roche, Ventana Medical Systems Inc.), and Iba1 (FUJIFILM Wako Pure Chemical Corporation, Osaka, Japan) for T cells, B cells, and microglia/macrophages identification, respectively. For each antibody, the number of positive cells was counted in five consecutive 40× fields in three brain macro-regions (pons/medulla oblongata, midbrain/thalamus, and cerebellum) starting from more densely stained areas. For immunofluorescence (IF) analyses with the confocal microscope, sections were re-hydrated and antigen retrieval was performed by incubation in citrate buffer 0.01 M pH 6 at 95°C for 20 min. Slides were then permeabilized for 20 min at room temperature with PBS 1% Triton X-100, saturated with Blocking Buffer (bovine serum albumin [BSA] 5% in PBS 0.1% Triton [PBSt]) for 1 h and incubated overnight at 4°C with the primary antibody. The following day, samples were incubated in the dark for 2 h, with the secondary antibody conjugated with a fluorophore previously diluted in 1% BSA in PBSt. Slides were washed and mounted in with Fluoroshield Mounting Medium with DAPI (Sigma) in order to label the nuclei of the cells, thus allowing a better visualization, interpretation and counting. Images were acquired with Leica TCS SP8 confocal microscope equipped with a CCD camera at 63× enlargement using LAS AF 2.7.3.9723 software and analyzed using ImageJ. For the investigation of the immunoglobulin (IgG) deposition in infected Syrian hamster brains, we counted the number of IgG-positive cells among at least 1,000 infected cells. Primary and secondary antibodies for IF and IHC are listed in Table S3.

### Molecular analyses for the quantification of the viral RNA

To quantify the viral RNA in the brain samples, we developed three species-specific quantitative real-time RT-PCRs using the AgPath-ID One-Step RT-PCR Reagents (Life Technologies) on a CFX96 Touch Deep Well Real-Time PCR Detection System (Bio-Rad). For each brain, approximately 25 mg of tissue was homogenized in RLT Lysis buffer (Qiagen) using the TissueLyser (Qiagen). Total RNA was isolated using the Rneasy Mini kit (Qiagen), and contaminant DNA was removed with the Rnase-Free Dnase Set (Qiagen). The host β-actin mRNA (51) was also amplified to assess the quality of the samples. Viral RNA copies/μL of total RNA were calculated using synthetic RNA standard curves prepared in negative brains RNA (ranging from 10^8^ to 10^3^ copies/μL), starting from samples having the same RNA concentration, in order to be able to compare them. Oligonucleotide and probes sequences are listed in Table S4.

### Gene expression analyses by RNA-sequencing

We investigated virus-host response by performing the brains transcriptomic profile of infected (with either DUVV, WCBV or RABV) versus mock animals. All the individuals were processed and analyzed. Analyses were preceded by a quality check of RNA in the samples using Agilent RNA 6000 Nano kit, considering as acceptable RIN values ≥8. Libraries were prepared from 500 to 1,000 ng of total RNA with the Truseq Stranded mRNA library preparation kit (Illumina), following the manufacturer’s instructions and were run on an Agilent 2100 Bioanalyzer using an Agilent High Sensitivity DNA kit (Agilent Technologies) to ensure the proper range of cDNA length distribution. Sequencing was performed on Illumina NextSeq with NextSeq 500/550 High Output Kit v2.5 (300 cycles; Illumina) in pair-end [PE] read mode producing about 33 million reads per sample. We filtered raw data by clipping the library adaptors and trimming low-quality ends with trimmomatic v0.391 (52) and we removed remaining reads shorter than 50 bp. After filtering raw data, we aligned high-quality reads Mesocricetus auratus (BCM Maur 2.0, NCBI) (53) using STAR v2.7.9a (54) and generated the gene count using htseq-count v0.11.0 (55). We then investigated the differential expression of genes between infected and mock Syrian hamsters with Deseq2 package v1.20.0 (56), using FDR < 0.05 and |log2FC| ≥ 1 and assigned gene ontology (GO) terms to each gene using Blast2GO v5.2.5 (57). Child-father relationships belonging to GO graph were reconstructed using the OBO file downloaded from http://geneontology.org/ (accessed on 19 October 2021). GO enrichment was computed by Fisher’s exact test and *P* values were adjusted through the Benjamini–Hochberg correction, considering as significant an FDR < 0.05 (Tables S5 and S6). Enrichment scores were computed as −log(FDR).

### Molecular analyses for gene expression confirmation

Real-time PCR was performed on retrotranscribed cDNAs with a two-step PCR approach, using Bio-Rad CFX thermal cycler and analyzed with Bio-Rad CFX Maestro software. cDNA synthesis was carried out with oligo(dT)-primed Superscript II (Invitrogen). Real-time PCR was performed using specific primers for the selected genes with each sample analyzed in triplicates; the dye-base detection and amplification of the target genes were achieved with the SsoFast EvaGreen Supermix (Bio-Rad). The target genes Ct (cycle threshold) were normalized to the β-actin housekeeping gene and the relative mRNA expression levels were evaluated as fold increase compared to the mean of the mock animals for each gene analyzed. Oligonucleotide sequences and relative targets are listed in Table S4.

### Statistical analyses

**Fig 1:**
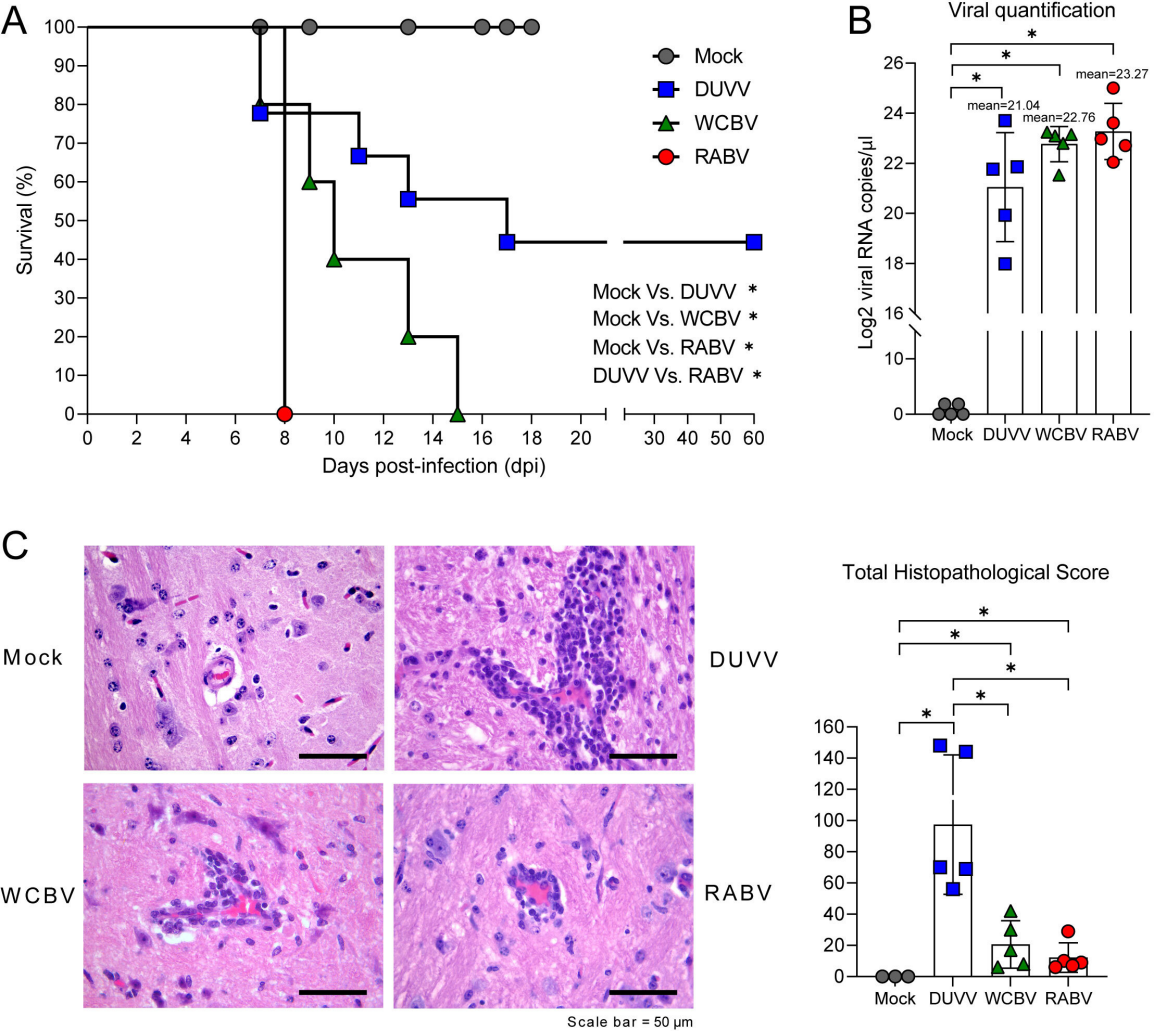
Lyssaviruses infection in the Syrian hamster model. (**A**) Percent survival over time in Syrian hamsters infected with either DUVV, WCBV or RABV. Kaplan–Meier survival curves are shown by plotting percent survival against time (days post-infection). Mantel–Cox test was performed to compare survival curves of the Mock group versus infected groups and DUVV versus WCBV versus RABV. (**B**) Lyssaviruses viral load as determined through quantitative real-time RT-PCR on infected brain samples; results are expressed as Log2 copies/µL of genomic RNA. All the symptomatic animals were analyzed. Mean values ± SD are represented. Wilcoxon–Mann–Whitney test of Mock group versus infected groups and DUVV- versus WCBV- versus RABV-infected hamsters. (**C**) (Left) Representative images of brains from infected and control (Mock animal, pons, animal no. M1, total histopathological score 0) Syrian hamsters; incomplete, mild (score 1) mononuclear perivascular cuffs from the pons of animals infected with WCBV (animal no. 6, total histopathological score 30) and RABV (animal no. 2, total histopathological score 9). Severe (score 3) perivascular cuff, composed of more than two cell layers, in the cerebellar white matter of an animal infected with DUVV (animal no. 12, total histopathological score 148). Scale bar = 50 µm. (Right) Final histopathological score of brain lesions for all the symptomatic animals; details on the scores are available in Table S2. Wilcoxon–Mann–Whitney test of Mock group versus infected groups and DUVV- versus WCBV- versus RABV-infected hamsters. For all the data presented, statistically significant comparisons are shown and represented with a asterisk (*). *P* values are listed in Table S7.

**Fig 2:**
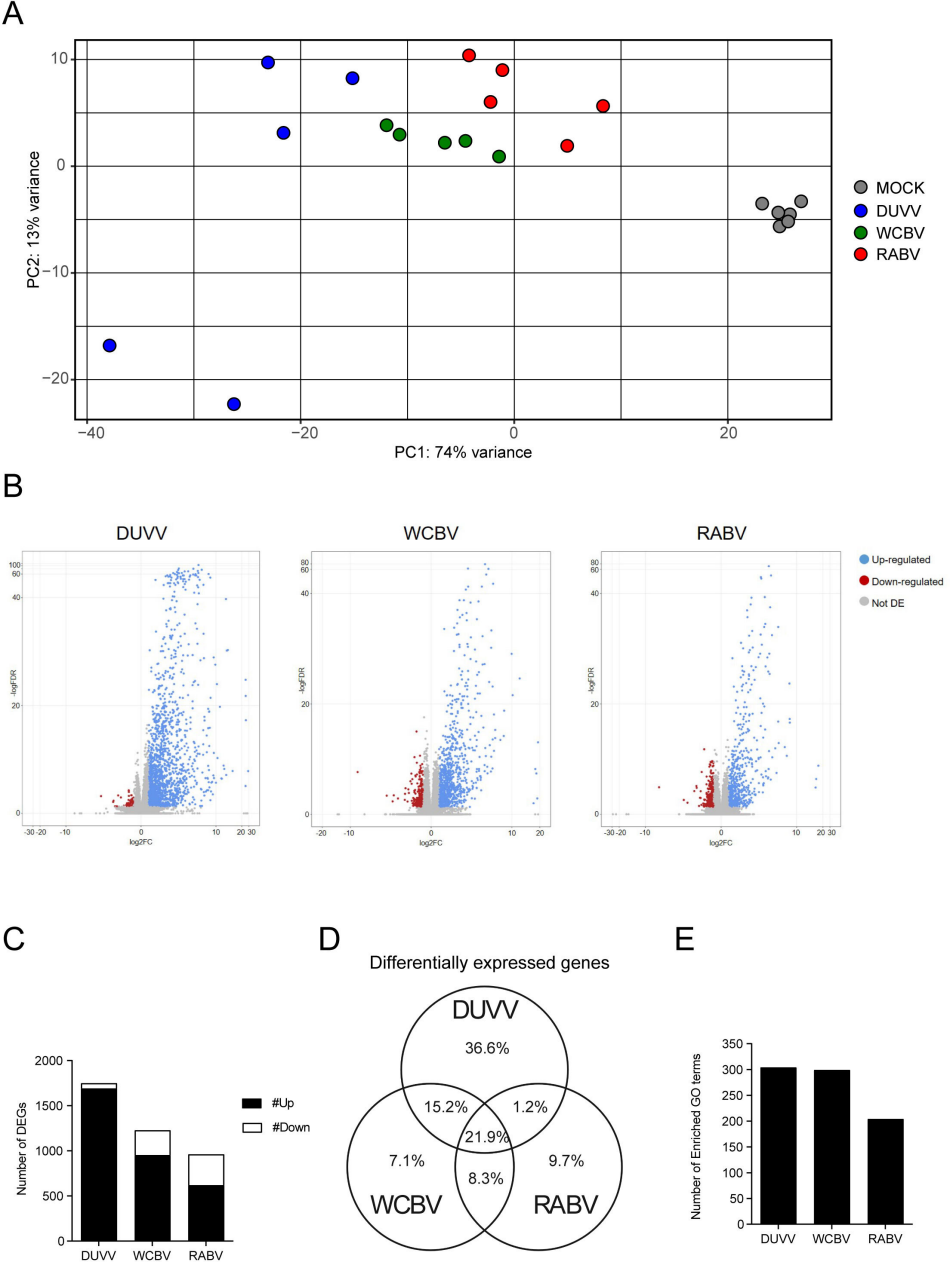
RNA-Seq expression profiles in DUVV-, WCBV-, and RABV-infected brains. (**A**) Principal component analysis (PCA) of the normalized gene counts of the host genes. The *X* and *Y* axes show the two dimensions that explain the overall amount of variance related to gene expression levels. Each replicate is represented by a colored code based on the challenge virus: Mock (gray), DUVV (blue), WCBV (green), and RABV (red). (**B**) Volcano plots showing differential expression analysis results for DUVV-, WCBV-, and RABV-infected brains (blue, upregulated; red, downregulated; and gray, not significant). A DEG was significant in a comparison when Log2FC ≤ −1 or Log2FC ≥ 1 and FDR < 0.05. (**C**) Total numbers of DEGs for each comparison of infected versus mock found in the differential expression analysis; up- and downregulated genes are respectively shown in black and white. See also Table S5 for the raw numbers of DEGs. (**D**) Venn diagram for the graphic representation of the percentages of DEGs associated with each lyssavirus and in common between two or all of the viruses. (**E**) Number of enriched GO terms for every comparison of infected versus mock found in the analysis. See also Table S6 for the raw numbers of DEGs.

**Fig 3:**
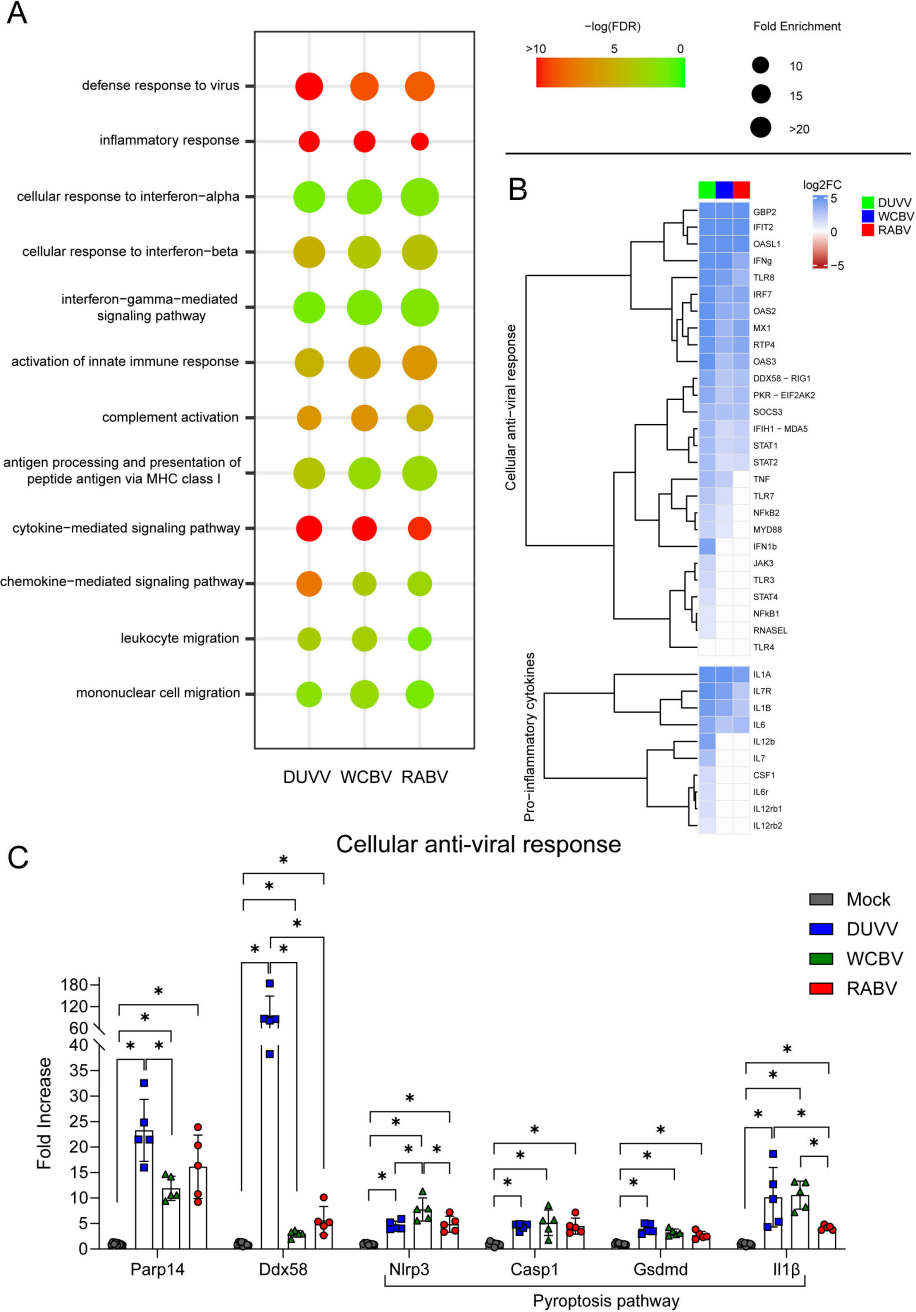
Transcriptomic profile of lyssaviruses-infected Syrian hamsters. (**A**) Dotplot representing the commonly enriched gene ontology (GO) terms related to immunity in the lyssaviruses-infected brains. Statistically significant enrichments (FDR < 0.05) are presented and the −LogFDR is shown. See also Table S6 for the raw data on the GO analysis. (**B**) Heatmap (Log2FC values of the infected vs mock comparisons) of selected genes related to the cellular anti-viral response and pro-inflammatory cytokines production. A DEG was significant in a comparison when Log2FC ≤ −1 or Log2FC ≥ 1 and FDR < 0.05. See also Table S5 for the raw data on the DEGs analysis. (**C**) Real-time PCR results. Relative mRNA expression levels were evaluated as fold increase compared to the mean of the mock animals for each gene analyzed. Bars represent mean ± SD and all the single values are also plotted. Wilcoxon–Mann–Whitney test of mock animals versus DUVV-, WCBV-, and RABV-infected animals and of DUVV- versus WCBV- versus RABV-infected hamsters. Only statistically significant comparisons are shown and represented with a asterisk (*). *P* values are listed in Table S7.

**Fig 4:**
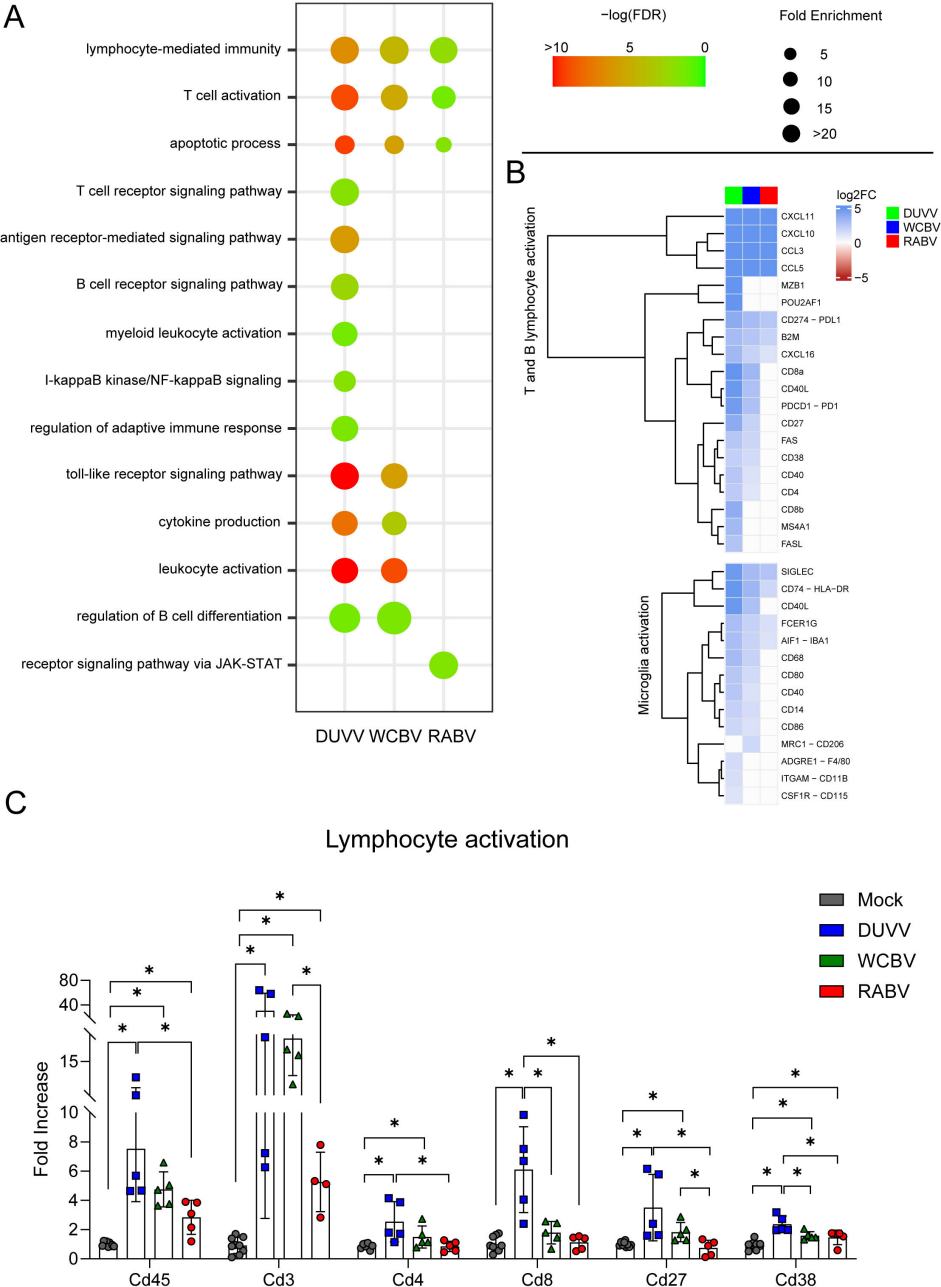
Transcriptomic analyses highlight non-RABV lyssaviruses as being strong activators of the adaptive immune response. (**A**) Dotplot representing the most specific enriched gene ontology (GO) terms related to immunity in the lyssaviruses-infected brains. Statistically significant enrichments (FDR < 0.05) are presented and the −LogFDR is shown. See also Table S6 for the raw data on the GO analysis. (**B**) Heatmap (Log2FC values of the infected vs mock comparisons) of selected genes related to the lymphocyte activation and microglia recruitment. A DEG was significant in a comparison when Log2FC ≤ −1 or Log2FC ≥ 1 and FDR < 0.05. See also Table S5 for the raw data on the DEGs analysis. (**C**) Real-time PCR results. Relative mRNA expression levels were evaluated as fold increase compared to the mean of the mock animals for each gene analyzed. Bars represent mean ± SD and all the single values are also plotted. Wilcoxon–Mann–Whitney test of mock animals versus DUVV-, WCBV-, and RABV-infected animals and of DUVV- versus WCBV- versus RABV-infected hamsters. Only statistically significant comparisons are shown and represented with a asterisk (*). *P* values are listed in Table S7.

All statistical analyses were performed using GraphPad Prism 10. For the comparison of the survival curves presented in Fig. 1A, we applied the Mantel-Cox (or Log-Rank) test. For the results presented in Fig. 1B and C, we applied the Wilcoxon–Mann–Whitney test for independent groups. We did not apply any statistical analysis of the data presented in Fig. 2. For the data shown in Fig. 3C, 4C, 5A through C and 6B, we applied the Wilcoxon–Mann–Whitney test for independent groups. For all statistics, we considered as significant *P* values < 0.05. Details on the statistical results are presented in Table S7.

## RESULTS

### WCBV is highly lethal and triggers mild encephalitis in Syrian hamsters

Upon intramuscular injection, RABV- and WCBV-infected Syrian hamsters all died at 8 and from 9 to 15 days post-infection (dpi), respectively. The first DUVV injection of five hamsters led to the actual development of rabies disease in three animals only. The *in vivo* procedure was then repeated on further four hamsters, with only two individuals productively infected; this second attempt led to five the total number of DUVV-infected animals developing rabies symptoms out of nine that had received the intramuscular injection (5/9; 55.55%) (Fig. 1A). Importantly, in all the symptomatic animals, the average level of viral genome present in the CNS, as evaluated by real-time RT-PCR and shown as copies/µL, was not statistically different among groups. Indeed, although animals infected with DUVV displayed more heterogeneity in viral titers compared to WCBV and RABV (DUVV mean Log2 copies/µL 21.04 [23.21–18.87]; WCBV mean Log2 copies/µL 22.76 [23.47–22.05]; RABV mean Log2 copies/µL 23.27 [24.39–22.15]), these differences were not statistically significant (Fig. 1B; Table S7). All WCBV-, RABV-, and DUVV-infected hamsters developed mild to severe encephalitis characterized by perivascular cuffs, minor inflammatory cell infiltration of the brain parenchyma, and minimal leptomeningeal involvement (Fig. 1C; Table S2). Inflammatory cells included lymphocytes, macrophages and few plasma cells. Perivascular cuffs and parenchymal infiltration were commonly associated with gliosis (Fig. 1C). Inflammatory changes were observed in the gray and white matter, and were mainly located in the pons, medulla oblongata, midbrain, thalamus, and the ventral cerebellum. WCBV-infected animals exhibited mild inflammatory changes, similar to those observed in RABV-infected animals. Animals infected with DUVV exhibited significantly higher scores compared to those infected with both WCBV and RABV. In WCBV- and RABV-induced encephalitis, perivascular cuffs were incomplete or composed of a single complete cell layer (score 1 and 2). Perivascular cuffs of two or more complete cell layers (score 3) were found in four out of five DUVV-infected animals (Fig. 1C; Table S2). No significant differences were observed in the scores among the three assessed macro-regions (pons/medulla oblongata, midbrain/thalamus, and cerebellum) for any of the viruses.

### RNA Seq analysis to evaluate lyssavirus-host interaction

We then compared the CNS’s expression profiles in infected versus mock animals for each virus under evaluation using RNA-Seq technology. A principal component analysis (PCA) was first used to explore the differences in the general transcriptomic profiles of the analyzed brains, limiting the analysis on the first two dimensions; that is, the ones showing the greatest variance among data (Fig. 2A). The PCA of the normalized counts for the Syrian hamster highlighted the first PCA component (PC1) having the greatest variance (74%), clearly dividing the mock animals from the infected ones. Moreover, RABV- and WCBV-infected animals appeared to be clustering together, while DUVV-infected animals were separated. Looking at the second PCA component (PC2; 13%), no significant results emerged except for the clear separation of two samples belonging to the DUVV infection, something that seems to be related to the experiment itself instead of the specific viral infection. In general, DUVV-, WCBV-, and RABV-infected brains seemed well clustered and separated from each other (Fig. 2A). Overall, all the three lyssaviruses showed high numbers of differentially expressed genes (DEGs), with DUVV-infected hamsters accounting for the highest number of DEGs (*n* = 1,749) compared to WCBV- (*n* = 1,227) and RABV-infected animals (*n* = 960) (Fig. 2B through D; Table S5). The majority of the DEGs were upregulated, with some differences between the three viruses in terms of percentage of up- and downregulated genes. In the DUVV infection, the vast majority of the differently regulated genes were overexpressed (>96%), while for WCBV and RABV about 77% and 65% of the total DEGs were upregulated, respectively (Fig. 2C). DUVV infection also harbors the highest number of specific DEGs (36.6%) in comparison to WCBC and RABV (7.1% and 9.7%, respectively). About 21.9% of the overall DEGs were in common with all the three lyssaviruses. The investigation of the enriched biological processes through GO analysis showed that both DUVV and WCBV were characterized by a similar number of enriched GO terms (304 and 299, respectively), despite DUVV promoting the different regulation of 40% more genes than WCBV (Fig. 2E; Tables S5 and S6). On the other hand, RABV infection resulted in 204 GO terms enriched, in accordance with a lower number of DEGs compared to the other investigated viruses (Fig. 2C through E).

### DUVV, WCBV and RABV promote a general cellular anti-viral state

Comparing the GO terms enriched by the lyssaviruses infection, we observed that many processes related to the host response to viral infection and to the activation of the innate immune response were commonly triggered by the three viruses under investigation. In this regard, functions as “activation of innate immune response,” “defense response to virus,” “interferon-gamma-mediated signaling pathway,” “complement activation,” and “leukocyte/mononuclear cell migration” were highly enriched by all the three lyssavirus infections (Table S6; Fig. 3A). Furthermore, we found that all viruses strongly activate the processes “inflammatory response” and “cytokine-mediated signaling pathway,” with DUVV showing the highest enrichment scores (enrichment scores: inflammatory response: DUVV 109,659; WCBV 83,060; and RABV 42,312; cytokine-mediated signaling pathway: DUVV 33,184; WCBV 16,497; and RABV 9,631 [Table S6; Fig. 3A]). Of note, while all the three lyssaviruses upregulated key genes associated with the cellular anti-viral response (e.g., *Ddx58*, *Mda5*, and *Mx1*), we observed a specific upregulation of other genes particularly related to the NFkB pathway (e.g., *Nfkb1-2* and *Myd88*) in DUVV-infected brains. Moreover, the three lyssaviruses under investigation promoted a higher expression of several pro-inflammatory cytokines (e.g., *Il1α*, *Il1β*, and *Il6*) compared to mock animals, while we observed that DUVV is able to specifically boost the expression of other pro-inflammatory cytokines-related genes such as *Il12b* or *Il7* (Fig. 3; Table S5). We then confirmed, through gene-specific real-time PCRs, the upregulation of selected genes associated with cellular anti-viral sensing (*Parp14* and *Ddx58*) and the activation of the inflammosome and pyroptosis (*Nlrp3*, *Gsdmd*, *Casp1*, and *Il1β*). Of note, DUVV-infected hamsters had the highest levels of *Ddx58* expression compared to WCBV and RABV and both DUVV and WCBV induced a greater expression of the pro-inflammatory cytokine *Il1β*, compared to RABV (Fig. 3C; Table S5).

### DUVV and WCBV stimulate immune cell recruitment in the CNS better than RABV

We then identified the biological processes that were different in the three infections, being specifically enriched by one single virus. Strikingly, DUVV and WCBV promoted a higher enrichment of “lymphocyte-mediated immunity,” “T cell activation,” and “cytokine production” processes compared to RABV (Fig. 4A). Specifically, DUVV-infected animals fostered “lymphocyte mediated immunity” and “T cell activation” stronger than the other two viruses (enrichment scores: DUVV 6.645; WCBV 4.462; RABV 2.230, and DUVV 8.990; WCBV 5.126; RABV 1.340, respectively, for the two GO terms) and exclusively enriched several biological functions such as “T and B cell receptor signaling pathway,” “antigen receptor-mediated signaling pathway” and “regulation of the adaptive immune response” (Table S6). Apart from the more classical cytokines produced during the inflammatory process (e.g., *Cxcl10-11* and *Ccl3-5*), we observed that many cytokine-related genes associated to lymphocyte activation were strongly up-regulated in DUVV- and, to a lesser extent, in WCBV-infected hamsters, with almost zero expression in RABV infection (e.g., *Cd40*, *Cd40l*, *Cd4*, *Cd8b*, *Cd27*, and *Cd38*); such a pattern was also evident for genes linked to the microglia activation [e.g., *Aif1*, *Fcer1g*, *Cd40*, *Cd68*, and *Cd80* (13, 58)] (Fig. 4B; Table S5). To strengthen the results obtained by the RNA-Seq analysis, we evaluated the expression levels of genes associated with lymphocyte activation by performing real-time PCRs and evaluating the fold increase in gene expression in infected versus mock animals (Fig. 4C). Syrian hamsters infected with DUVV and WCBV promoted the highest expression of genes related to lymphocyte differentiation (*Cd45*), T- (*Cd3*, *Cd4*, and *CD8*) and B-lymphocyte activation (*Cd27* and *Cd38*) compared to RABV animals. Of note, two DUVV-infected hamsters showed the greatest expression levels in particular of the B-lymphocyte-associated genes, with the remaining three genes having an expression comparable or slightly above WCBV animals (Fig. 4C). The obtained results confirmed the increased lymphocyte involvement in the CNS during a DUVV and a WCBV infection.

### DUVV stimulates lymphocyte-mediated immunity in the CNS

**Fig 5:**
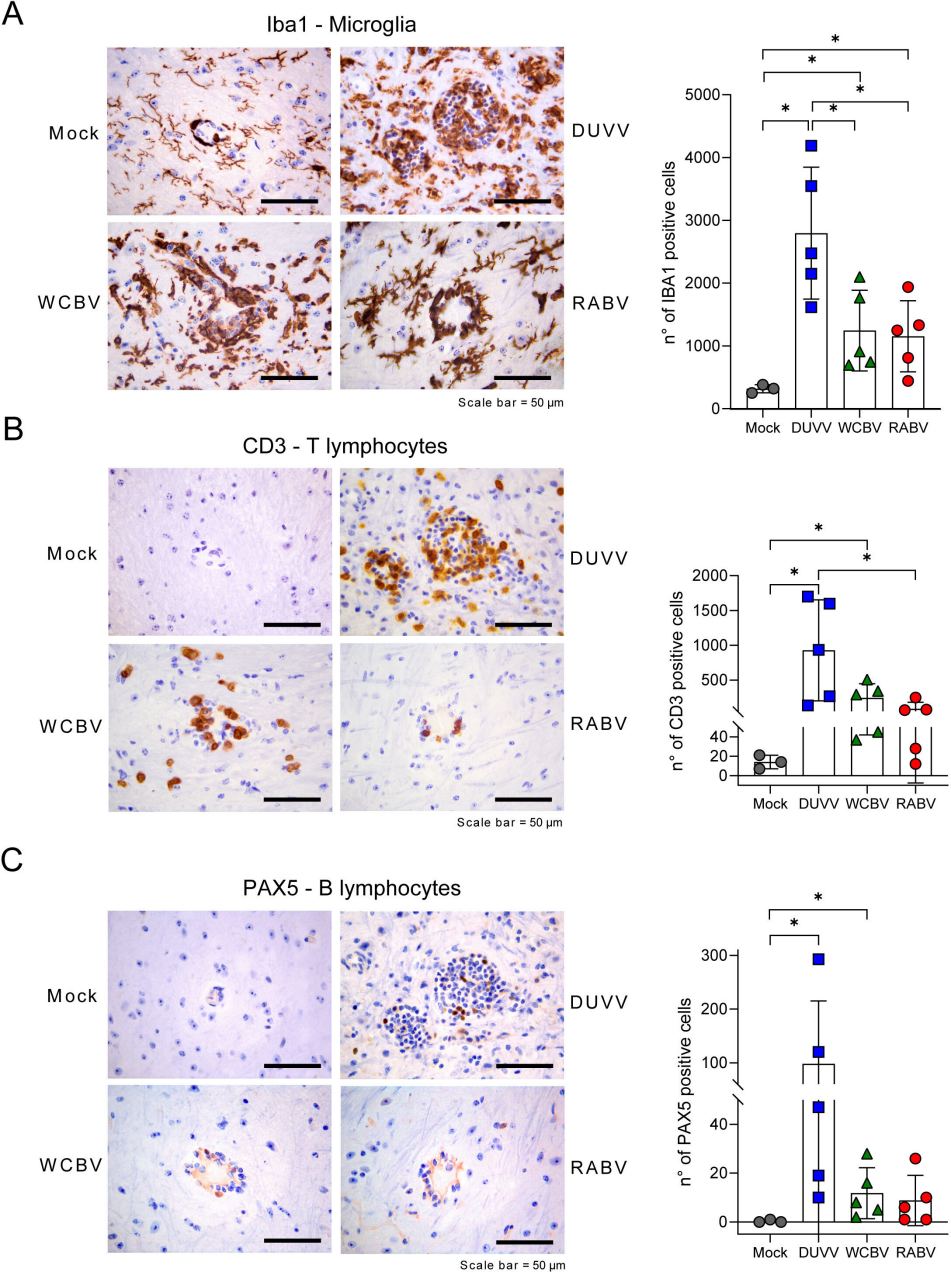
Differential immune recruitment in the lyssaviruses-infected brains. Representative IHC staining (left) for the microglia/macrophage marker Iba1 (**A**), the T lymphocyte marker CD3 (**B**) and the B lymphocyte marker PAX5 (**C**), representative images of the pons (left). Scale bar = 50 µm. Counts of Iba1- (**A**), CD3-, (**B**) and PAX5-positive cells (**C**) are also shown (right); mean values ± SD are representative of mock group versus infected groups and DUVV- versus WCBV- versus RABV-infected hamsters are shown. Statistically significant comparisons are shown and represented with a asterisk (*). *P* values are listed in Table S7. All animals were analyzed; representative images were shown.

**Fig 6:**
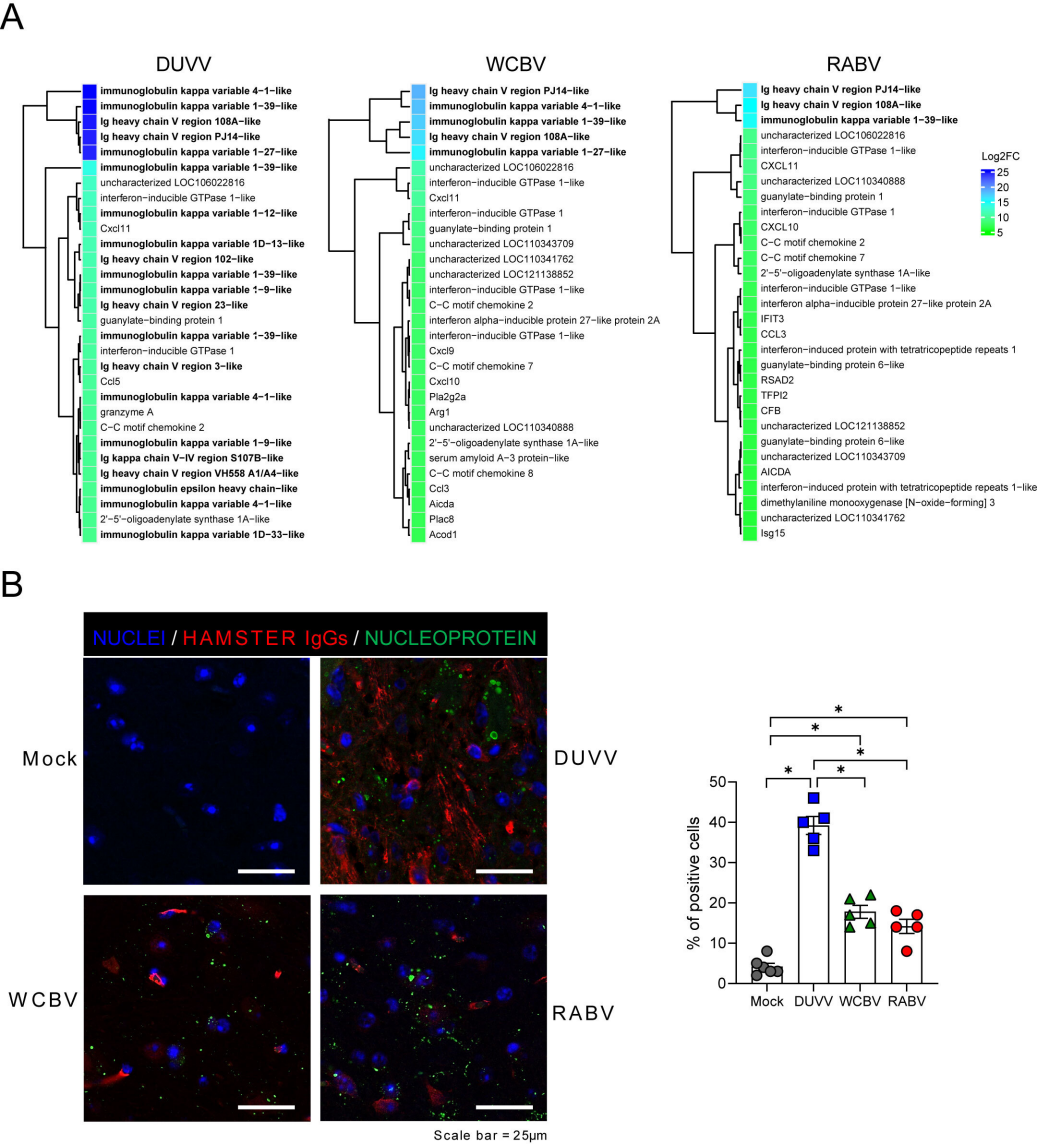
DUVV virus foster immunoglobulin deposition in the CNS. (**A**) Heatmap (Log2FC values of the infected vs mock comparisons) of the top 30 DEGs in the DUVV-, WCBV-, and RABV-infected brains. (**B**) Immunofluorescence images (left) and relative quantification (right) for hamster immunoglobulin (red) deposition in the infected CNS, coupled with viral nucleoprotein (green) staining and nuclei (blue). Scale bar = 25 µm. All animals were analyzed; representative images were shown. Bars represent mean ± SD of the percentage of positive cells, and all the single values are also plotted. Wilcoxon–Mann–Whitney test of mock animals versus DUVV-, WCBV-, and RABV-infected animals and of DUVV- versus WCBV- versus RABV-infected hamsters. Only statistically significant comparisons are shown and represented with a asterisk (*). *P* values are listed in Table S7.

To characterize the inflammatory infiltrate, IHC for CD3 (T lymphocyte), PAX5 (B lymphocyte) and Iba1 (microglia/macrophage) was conducted. Perivascular cuffs were mainly composed of T lymphocytes, macrophages, and a lesser number of B lymphocytes (Fig. 5A and B). The number of infiltrating T, B lymphocytes and Iba1-positive microglia/macrophages increased with the severity of histopathological changes. In particular, counts of T cells and microglia/macrophages were significantly higher in DUVV-infected animals than in RABV-infected ones (Fig. 5A and B). Moreover, DUVV-infected animals exhibit higher macrophages/microglia counts compared to WCBV-infected animals (Fig. 5A). It is important to note that, despite a substantial variability among subjects, DUVV-infected hamsters displayed high levels of PAX5-positive cells, suggesting a higher involvement of the B cells compartment. Indeed, one of the crucial aspects of the establishment of lymphocyte-mediated immunity is the differentiation of B lymphocytes and their commitment to the production of specific antibodies against the virus (59, 60). Interestingly, we noticed that 21 out of top 30 DEGs were upregulated immunoglobulin fragment-expressing genes in DUVV-infected hamsters, corroborating a stronger engagement of antibody-producing B cells in the CNS in response to the infection with this lyssavirus (Fig. 6A; Table S5). To confirm what observed as DEGs, we evaluated the presence of IgG immunoglobulins presence (61–63) in the brain of infected and mock animals and confirmed that DUVV had the strongest ability to foster immunoglobulin (Ig) deposition, although the three infections all promoted Ig recruitment in the CNS differently from what observed in mock animals (Fig. 6B).

## DISCUSSION

**Fig 7:**
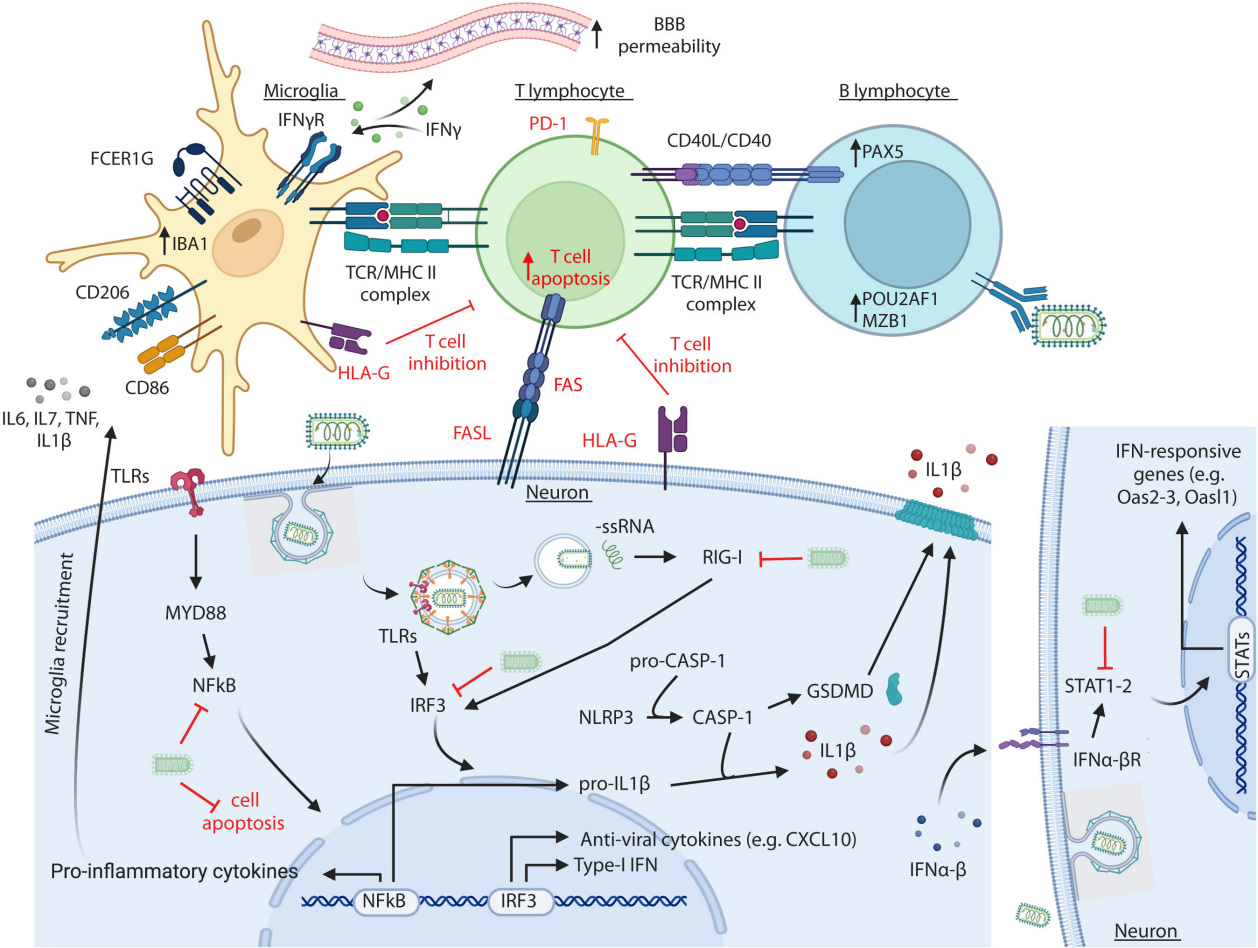
Immune response against RABV in the CNS. Cartoon representing the main cellular pathway and processes that are either activated or inhibited during RABV infection in the CNS. Highlighted in red are the mechanism by which high pathogenic RABV strains blunt the anti-viral and immune response of the host (1, 5, 13–15, 28–30, 32, 33, 35, 36, 42, 58, 60, 62, 64–67, 69–72, 75–98). Created with Biorender (agreement number AG27HPXNB3).

Although not entirely understood, RABV pathogenesis has been extensively addressed. The main aspect of this viral infection is the impairment of both immune and brain functions, which almost invariably results in fatal outcomes without significant histopathological lesions in the CNS (64). Indeed, RABV is able to antagonize and block several players of the innate host response to RNA viruses (14, 15, 36). More specifically, RABV proteins prevent viral recognition by the host cell through the interaction with specific cellular elements, such as the cytosolic ss-RNA sensor RIG-I (65, 66). RIG-I induces the downstream expression of antiviral genes (e.g., *CXCL10*) (67, 68) and promotes the entry of the NFkB complex into the nucleus with the activation of the antiviral responses including apoptosis (69). Regarding the recruitment of the NLRP3 inflammosome and the activation of the inflammation-promoting pathway known as pyroptosis, involved in the inflammatory response during different viral infections (70–72), the expression of the key elements of this process (*Gsdmd*, *Casp1*, and *Il1β*) differ in relation to the strain pathogenicity (14, 15, 30, 73–75). In particular, RABV pathogenic strains cause mild inflammation in the CNS, while attenuated strains determine severe inflammation and neuronal death (15, 36, 76, 77) (Fig. 7).

In terms of histopathological findings, only few infiltrating cells are detected in the brains of animals infected with highly pathogenic strains. On the contrary, low pathogenic/attenuated strains strongly activate the innate and adaptive response in the CNS, with the recruitment of mononuclear cells and lymphocytes thanks to the release of the IFNs that eventually increases the blood-brain barrier permeability (32, 35, 36, 78–82). It is worth noting that the infection with pathogenic RABV strains results in impaired lymphocyte-mediated response during encephalitic fatal infection as T cells are destroyed by apoptosis in the CNS, whereas ingection with attenuated RABV strains leads to the induction of Th1-type immune response and initial control of viral replication (33). Together with the cellular antiviral response, humoral immunity plays an important role in the elimination of RABV in the CNS. Indeed, the secretion by activated B cells of virus neutralizing antibodies cooperating with dendritic cells and Th1 lymphocytes to clear the virus, is more often associated with attenuated strains (35, 83, 84) (Fig. 7).

In the present study, we analyzed the histological changes in the brain and uncovered the immune response elicited by the Italian WCBV in an accidental host, specifically female Syrian hamsters. We chose to select only female animals in order to avoid any sex-biased difference, as many recent works have clearly demonstrated that immune response against viruses might change between males and females (99–102). The WCBV strain under investigation was detected in a domestic cat in 2020 and subsequently passaged twice in newborn mice, as the other lyssaviruses under examination. For the purpose of our study, viral stocks were amplified through IC inoculation in newborn mice to minimize the occurrence of mutations that are rather frequent during lyssavirus *in vitro* replication (103–105). Indeed, the mouse-passaged WCBV viral stock fully retained the genetic makeup of the original cat-derived strain MZ501949 (Table S1). The transcriptomic profile and brain lesions triggered by WCBV were compared to those determined by two lyssaviruses used as standards, namely a highly pathogenic RABV and DUVV, a lyssavirus characterized by low pathogenicity (41, 42), and by using Syrian hamsters injected with the viral diluent as Mock animals. We exploited the intramuscular (IM) route that is well-known in the pathogenesis of RABV and constitutes the most likely natural route of infection also for the entire genus. In addition, contrarily to the intra cerebral (IC) or intra nasal (IN) routes, the IM infection offers the unique opportunity of evaluating the viral ability to reach the CNS (neuroinvasiveness).

First, our data confirm the selected strains of RABV and DUVV as being the correct standards for high and low pathogenicity, respectively. Indeed, while RABV caused 100% lethality, DUVV determined the death of only 55% of hamsters (five out of nine) in our experiment, corroborating previous evidence showing low neuro-invasiveness and pathogenicity in mice through IM infection (41). In this context, it is worth noting that previous studies determined the intramuscular pathogenicity index of DUVV as being the lowest among 13 lyssaviruses (41). In our experiment, WCBV was able to invariably reach the hamsters’ CNS, where it promoted a productive infection and fatal outcomes in 100% of the infected animals (five out of five hamsters developing rabies signs), mirroring the infection with highly lethal RABV. The data are consistent with previous preliminary evidences obtained with the original isolate in the same animal model at a similar dose (106). Sequence comparison of the two WCBV strains displayed few aminoacidic changes in the P (3), M (1), G (1), and L (7) genes (Table S1) (49). Among these non-synonymous mutations, V293I (P gene)—although not investigated yet—is worth of note as the C-terminal domain of the P protein seems to play a paramount role in blocking the JAK-STAT pathway and the IFN response (107, 108). The other aminoacidic changes found in the P sequence (L151F; N155S), particularly in the nuclear localization signal region, have not been identified as crucial residues for P activity. We also observed a single mutation in the G protein sequence (H496Q) located in the cytoplasmic domain and, so far, not associated with viral pathogenesis and/or cellular anti-viral response. Finally, several mutations occurring in the L protein sequence were identified: A25T; R115K; V287I; R347K; V1336A; S1411G; and S1787F. The role of these specific mutations is unknown, although we expect that they might be related to the main functions of the viral polymerase (L), such as promoting viral genome replication, mRNA transcription and viral propagation by interacting with other viral proteins (N and M) (1, 65, 109), (Table S1). In the future, reverse genetics would help us to understand the significance of the few aminoacidic changes observed in pathogenicity and ability to host jump. However, the identification of more WCBV strain representatives remains crucial to know whether the mutations observed had been acquired during the viral-host co-evolution or following spillover on the cat. As for DUVV, whole genome sequencing indicates few aminoacidic changes in the N (1), G (7), and L (2) sequences with the bat reference sequence EU293120.1. We identified mutation E127D in the N protein and individuated mutations A1392V and S2073A in the L sequence; none of them are connected with the pathogenicity of lyssaviruses strains. It is interesting to observe that the G protein sequence retains several variations compared to the original bat isolate, suggesting they might have acquired mutations while adapting to a new host. None of the amino acid substitutions have been reported yet (I23F; M172I; F173V; P174S; K177R; I310L; and L391F), but it is interesting to note that residues 173, 177, and 391 differ from the original bat isolate but are similar to RABV, further corroborating the assumption that these specific mutations might have a crucial role in the viral host adaptation.

WCBV appeared to behave similarly to the highly pathogenic RABV at a histological level, determining mild to moderate brain lesions in the affected hamsters. Indeed, our data confirm that DUVV induces great inflammatory changes, similar to other non-RABV species, including Lagos bat virus, Mokola virus, and European bat 1 and 2 lyssaviruses (40, 41, 51, 110, 111). Overall, our results support the inverse correlation postulated elsewhere between the lethality of lyssaviruses and the severity of encephalitic lesions (64).

Considering inflammation, RABV and DUVV infections expectedly determined the lowest and the highest ability to promote exogenous RNA sensing response (112) (e.g., TLRs genes, *Myd88*, *Mx1*, and *Mda5*), IFN response (e.g., *Stat1-2-4*, *Oas2-3*, and *Oasl1*) and pro-inflammatory cytokines production (e.g., *Il6*, *Il12b*, and *Il12rb1-2*), respectively (113). Of note, we observed that the cellular antiviral sensing mechanism (*Parp14* and *Ddx58* genes) was consistently overexpressed in DUVV-infected animals, confirming DUVV as an efficient promoter of the anti-viral cellular machinery. Indeed, the expression levels of specific genes involved in the cellular response to WCBV infection were similar to what observed following the infection with RABV (e.g., *Ddx58*, *Inf1β*, *Tlr3-4*, and *Nfkb*). This finding is consistent with the high lethality observed for WCBV and RABV. Pyroptosis appeared to be more pronounced in response to DUVV and WCBV than RABV infection. Our observations regarding the activation of pyroptosis are consistent with the comparative transcriptomics work by Koraka et al. in mice (42). Of interest, WCBV retained the ability of promoting pyroptosis similarly to DUVV, with a substantial over expression of both *Nlrp3* and ultimately *Il1β*.

Interestingly, the pro-inflammatory cytokines and chemokines involved in the microglia recruitment (e.g., *Il1b*, *Il6*, *Il7*, and *Tnf*,) and markers of an active state (e.g., *Cd68*, *Cd86*, *Fcer1g*, and *Cd206*) (58, 85) were abundantly upregulated by DUVV and poorly regulated by RABV. T and B cell recruitment and lymphocyte-mediated immunity were absent in RABV-infected brains while highly promoted by DUVV infection. WCBV fell in between the two extremes, thus retaining a moderate capacity to promote glial activation, and to establish an active pro-inflammatory environment. Based on RNA-seq results and the expression of marker genes (e.g., *Cd3*, *Cd4*, and *Cd45*), WCBV triggered T lymphocyte recruitment in the CNS, but poorly stimulated B cell activation [e.g., *Pou2af1* and *Mzb1*; (86, 114)]. Indeed, these findings were corroborated by IHC on brain sections, indicating a moderate increase of Iba1^+^ (microglia/macrophages) and CD3^+^ (T) cells and the low recruitment of PAX5^+^ (B) cells in WCBV-infected animals. The different extents of B-cell activation among the three viruses were also demonstrated by looking at the DEG expression levels and at the actual immunoglobulins’ (Igs) presence in the infected brains. Indeed, although Ig-related genes’ expression and Igs deposition were found to characterize the three infections, DUVV displayed the strongest ability to foster B-cell priming and IgGs deposition. More specifically, immunoglobulin’s fragment-related genes were the highest expressed DEGs during DUVV infection and both transcriptomic and real-time PCRs data showed high expression levels of activated B cells genes [e.g. *Cd27*, *Pou2af1*, and *Mzb1*; (86, 114)]. Considering the high differences in terms of pathogenicity between DUVV, WCBV and RABV, we speculate that the high stimulation of humoral immunity might explain DUVV low pathogenicity, leading to a more frequent viral clearance. In this context, the early presence of virus-neutralizing antibodies is considered a common finding and positive predictive factor in all the rare cases of recovery from RABV encephalitis, with titers dramatically increasing in response to the infection at both the blood and the cerebrospinal fluid (CSF) (115). It is also important to mention the complete recovery of mice intrathecally administered with RABV-neutralizing monoclonal antibodies (116).

Altogether, these latest findings indicate that DUVV acts similarly to what is described for low pathogenic RABV strains, with the activation of T lymphocytes and the maturation of B cells to secrete antibodies that, along with microglia activation and macrophage recruitment, contribute to the inflammatory state at tissue level and to the observed histopathological lesions that are representative of the attempts mediated by the immune system to eliminate the infection. Instead, WCBV seems to exhibit an intermediate phenotype between DUVV and RABV, promoting both a discreet activation of the anti-viral pathways and the recruitment of the innate and adaptive immunity; these, however, are not efficient weapons to reduce its pathogenicity.

A final consideration should be made on the activation of apoptosis, a process that appears enriched in WCBV- and, especially, in DUVV-infected hamsters. The induction of the programmed cell death promoted by RABV is still an open issue in the scientific community. What is currently known is that RABV preserves (i) the infected neurons and (ii) the cellular machinery using anti-apoptotic strategies, blocking any cytopathic mechanisms and fostering instead T cell death to prevent the clearance of the virus from the tissue without any traceable histopathological lesion (77, 87, 88). Moreover, this behavior seems typical of highly pathogenic strains, while attenuated/low pathogenic strains appear to promote high levels of apoptosis of the infected cells (89). The obtained data suggest that, with our experimental infections and bulk transcriptomic analyses, what we observe is the activation of apoptosis in cells infected with lyssaviruses rather than the programmed death of immune cells. Therefore, the genes that are widely known for promoting apoptosis in lymphocytes (e.g., *Fas*, *FasL*, and *Pdcd1*) are not upregulated in RABV, differently from what might be expected. However, we observe their increased expression in WCBV, even more in DUVV, outlining a scenario in which the highest promotion of inflammation and injury increases both the death of infected cells and of the lymphocytes that are massively recruited on site (Table S5). Further studies will be necessary to better investigate and characterize the role of this fundamental cell death process in the context of lyssaviruses infection.

In conclusion, our study provides the first CNS immunological characterization of the Syrian hamster model infected with WCBV, through molecular and immunological investigations. Although preliminary, our investigation paves the way toward a more comprehensive understanding of the mechanisms behind the immune response elicited by such a divergent lyssavirus and the possible significance in terms of host-jump and adaptation to new ecological niches. Altogether our results suggest that lyssavirus pathogenicity is conversely commensurate with the immune response triggered by the infection. Interestingly, WCBV was shown to be as lethal as RABV although it is somehow placed between RABV and DUVV in terms of brain lesions and ability to promote innate (monocytes/microglia), adaptive (lymphocyte) immune cell recruitment and cell activation in the CNS. The wide circulation of WCBV in its reservoir host (117), the bent-winged bat in the Palearctic region, coupled with the increased opportunity for human encroachment with wildlife (46, 118), are all issues that raise concern about the possibility for this virus to determine with increasing frequency a fatal disease in accidental hosts. In this context, it is worth noting that current biologicals available for RABV prophylaxis are supposed to be only partially efficacious in protecting both domestic animals and humans against viral infections caused by non-RABV lyssaviruses, with almost no protection against divergent lyssaviruses belonging to phylogroup III (119, 120).

### Limitations of the study

This study is based on the experimental results obtained by a single West Caucasian bat virus strain. The authors acknowledge that comparing multiple WCBV strains would have contributed to a greater understanding of the pathogenicity of this virus. The cat isolates under study (MZ501949) is the second ever isolate. Unfortunately, the reference strain (EF614258) isolated from the reservoir host (*M. schreibersii*) was not available as original brain material nor as a low-passaged batch in mice, useful for animal experiments. In this context, we cannot exclude that the observed findings are related to the strain itself rather than to a viral species peculiarity. Indeed, further experiments are foreseen to unveil the activation of this inflammation-promoting pathway by WCBV, also comparing the one elicited by strains isolated from the natural hosts versus the accidental one, and cell-adapted versus mice-passaged.

Considering the viral batches used within this work, they were not purified before injection and possibly contained a moderate amount of non-viral antigens that could be recognized by the host at the site of the inoculum. Injecting the UV-attenuated-mice brain homogenate as a negative control might also have provided additional information about the antigenicity of the inoculum, including the ability of the viruses to activate an immune response without replicating once injected peripherally (i.e., mimicking a vaccination). However, testing further hypothesis was well beyond the aim of the present study, whose objective was to compare the immunological patterns of the three encephalitis once the animals developed rabid signs. In this context, the differences observed clearly demonstrated that the viruses under investigation have a different pathogenicity and ability to stimulate the host immunity, although further experiments are needed to explore more specific hypotheses.

The findings of this work clearly demonstrate the different abilities of the lyssaviruses under examination to interact with the host and promote the activation of an immune response. It is worth explaining that, in this context, using a parasagittal brain section for the histological and immunohistochemical evaluations may represent a limitation, as it might not fully represent the overall inflammatory process. Therefore, further systematic analyses of brain regions as well as the neuroanatomical localization of the virus are necessary to better characterize WCBV-induced encephalitis. Moreover, our experiments did not include the collection of sera or CSF at different time points during the infection, also considering that deposition of Igs during lyssavirus infection had never been reported in previous literature. In this regard, the authors acknowledge that collecting this data could have helped to disentangle the actual role of humoral immunity in response to and possibly outcome of different lyssavirus infections and envisage this investigation to be carried out as part of further experiments.

## Data Availability

The RNA-seq data generated in this study have been deposited in the SRA database under the accession number PRJNA1068065. Full genome sequences of DUVV, WCBV and RABV are available under GenBank accession numbers PP869293, MZ501949.1, and OQ787037, respectively. Source data for the RNA-Seq analysis (Fig. 2 and 3, Fig. 4 and 6) and for histology, immunohistochemistry, and immunofluorescence (Fig. 1 and 5 and Fig. 6) are provided as supplemental material and are available from the corresponding author upon request.

## SUPPLEMENTAL MATERIAL

Supplemental legends jvi.01914-24-s0001.docx Legends for Tables S1 to S7.

Table S1 jvi.01914-24-s0002.xlsx Viral sequence alignments.

Table S2 jvi.01914-24-s0003.xlsx Histopathological scores.

Table S3 jvi.01914-24-s0004.xlsx Antibodies for IF and IHC.

Table S4 jvi.01914-24-s0005.xlsx Oligonucleotide sequences.

Table S5 jvi.01914-24-s0006.xlsx Differentially expressed genes.

Table S6 jvi.01914-24-s0007.xlsx GO processes.

Table S7 jvi.01914-24-s0008.xlsx Statistical results.

ASM does not own the copyrights to Supplemental Material that may be linked to, or accessed through, an article. The authors have granted ASM a non-exclusive, world-wide license to publish the Supplemental Material files. Please contact the corresponding author directly for reuse.
