# Supplementary material for: Immunological findings of West Caucasian bat virus in an accidental host
Source: J Virol. 2025 Jan 23;99(2):e01914-24. doi: 10.1128/jvi.01914-24 (PMC11853057; doi:10.1128/jvi.01914-24)
Supplement: Supplemental legends — Legends for Tables S1 to S7. [file jvi.01914-24-s0001.docx]

***Supplementary Material***

Immunological findings of West Caucasian bat virus in an accidental host

**Martina Castellan*^1^, Gianpiero Zamperin^2^, Greta Foiani^3^, Maira Zorzan^1^, Maria Francesca Priore^1^, Petra Drzewnioková^1^, Erica Melchiotti^3^, Marta Vascellari^3^, Isabella Monne^2^, Sergio Crovella^4^, Stefania Leopardi^1^ & Paola De Benedictis*^1^**

^1^Laboratory for Emerging Viral Zoonoses, WOAH Reference Laboratory for Rabies, FAO and National Reference Centre for Rabies, Department for Research and Innovation

^2^Viral Genomics and Transcriptomics Laboratory, Department for Research and Innovation, Istituto Zooprofilattico Sperimentale delle Venezie, 35020 Legnaro, Italy

^3^Laboratory of Histopathology, Istituto Zooprofilattico Sperimentale delle Venezie, 35020 Legnaro, Italy

^4^Biological Science Program, Department of Biological and Environmental Sciences, College of Arts and Sciences, Qatar University, Doha P.O. Box 2713, Qatar

*** Correspondence:**

Martina Castellan (MC)

[mcastellan@izsvenezie.it](mailto:mcastellan@izsvenezie.it)

Paola De Benedictis (PDB)

[pdebenedictis@izsvenezie.it](mailto:pdebenedictis@izsvenezie.it);

## SUPPLEMENTARY TABLES

**Table S1**. Genetic comparison between the WCBV and DUVV batch produced in new-born mice and the reference sequences. The amino acids count starts from the first methionine of the protein sequence.

**Table S2**. **a.** Histological scoring system for perivascular cuffs; summary of perivascular cuff scores, final histopathological scores, and CD3-, PAX5-, Iba1-positive cells counts. **b.** Histopathological scores and IHC cells counts; raw data.

**Table S3**. List of primary and secondary antibodies used for immunofluorescence (IF) and immunohistochemistry (IHC) and details of IHC protocols.

**Table S4**. List of real-time PCR and quantitative real time RT-PCR primers/probes used in the present work.

**Table S5**. Numbers of DEGs and Log2FC values found for each comparison made in differential expression analysis for the DUVV-, WCBV- and RABV- infected brains.

**Table S6**. Numbers of significant enriched GO terms and their scores found for each comparison made in differential expression analysis for the DUVV-, WCBV- and RABV- infected brains. GO terms are presented as blocks of the most specific term (i.e. the ones with the highest level) and their parent terms, based on the child-father relationships characterizing the GO graph.

**Table S7**. Table representing the statistical results referred to Figure 1A-C, Figure 3C, Figure 4C, Figure 5A-C and Figure 6C.
